# Supplementary material for: Axonal Regrowth of Olfactory Sensory Neurons In Vitro
Source: Int J Mol Sci. 2023 Aug 16;24(16):12863. doi: 10.3390/ijms241612863 (PMC10454582; doi:10.3390/ijms241612863)
Supplement: Supplementary file 1 [file ijms-24-12863-s001.zip › ijms-2548855-supplementary.pdf]

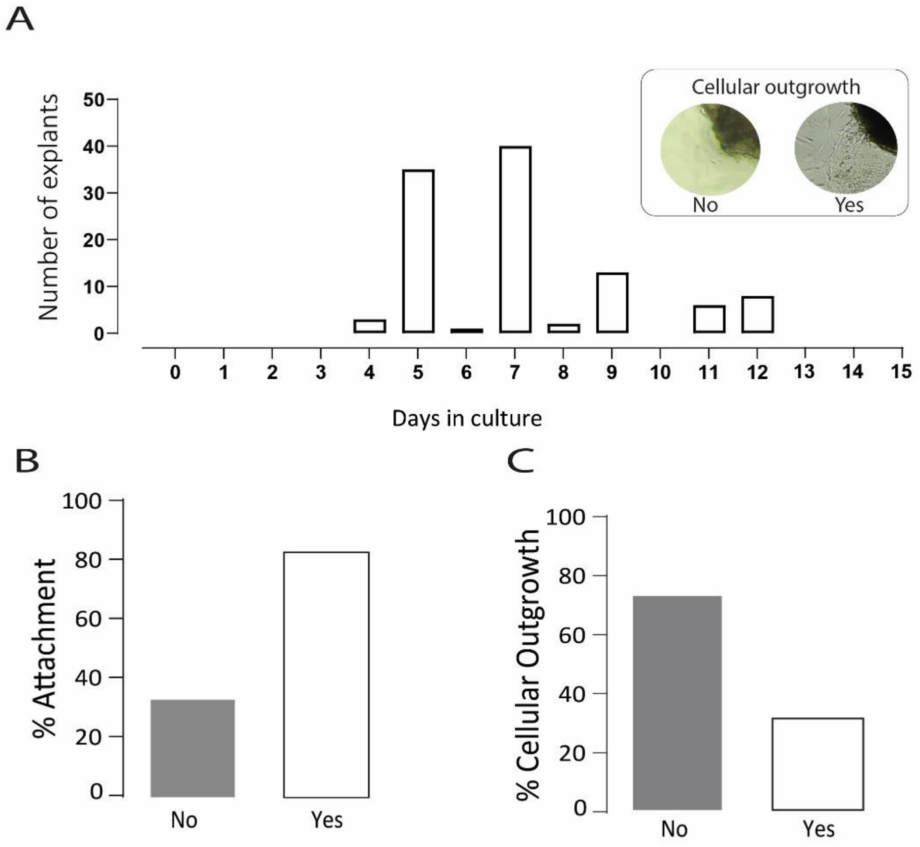

Supplementary Figure S1. Organotypic model settings. A The figure depicts the monitoring of proliferation rates in olfactory epithelium biopsies performed to achieve consistent cellular outgrowth frequency among the explants. Bright-field microscopy was used to visualize the first day of cellular outgrowth from the explants. The number of explants exhibiting signs of cellular outgrowth was recorded daily throughout the culture period. From day 7 to day 9, a window of potential days for introducing a differentiation growth factor was observed. **B and C** present the attachment and cellular outgrowth rates before fine-tuning anatomical harvesting. The attachment rate reached 84.9%, indicating successful adhesion of the explants to the coated wells. The cellular outgrowth rate, on the other hand, reached 35.8%, indicating the proportion of explants displaying cellular outgrowth. Descriptive statistics were performed using SPSS on an in vitro model involving 53 explants that were plated on coated wells.
